# Urinary schistosomiasis among preschool-aged children in Sahelian rural communities in Mali

**DOI:** 10.1186/1756-3305-4-21

**Published:** 2011-02-21

**Authors:** Abdoulaye Dabo, Haroun Mahamat Badawi, Boubacar Bary, Ogobara K Doumbo

**Affiliations:** 1 Department of Epidemiology of Infectious Diseases, Faculty of Medicine, Pharmacy and Dentistry, University of Bamako. Box 1805, Bamako, Mali; 2 Department of Pharmaceutical and Biomedical Sciences, University Institute of Sciences and Techniques of Abeche, (IUSTA), BP: 6077 N'Djamena, Chad

## Abstract

**Background:**

Mass chemotherapy with praziquantel is the main control strategy for schistosomiasis in Mali. However, in the national control programme for schistosomiasis and soil-transmitted helminthiasis, infants and preschool-aged children are overlooked in preventive chemotherapy campaigns. We therefore determined the prevalence and intensity of urinary schistosomiasis in children between the ages 1-4 years in three villages across Diema health district, a rural community with endemic schistosomiasis in Mali. For *Schistosoma haematobium *diagnosis, a single urine sample of 10 ml obtained from each child was subjected to the standard urine filtration method.

**Results:**

Of the 338 children examined 173 (51.2%) were infected. Both prevalence and intensity of infection varied significantly between communities (p < 0.01). There was no significant difference (p = 0.94) in infection rates between boys (51.2%) and girls (50.3%). Likewise, prevalence did not significantly increase with age (p = 0.86). The overall geometric mean of Williams (GMw) was 18.41 eggs/10 ml urine, with no significant association (p = 0.91) between boys (17.48 eggs/10 ml urine) and girls (19.69 eggs/10 ml urine). However, the GMw significantly increased with age (p = 0.04). Infection of preschool children would occur through early exposure to infected water bodies through both passive and active process.

**Conclusion:**

Our study showed that preschool children living closely to lakes across in Mali are at high risk to be infected by schistosomiasis and contributed largely to the transmission; therefore schistosomiasis control interventions should also target infants in addition to school children and adults in endemic areas.

## Background

Schistosomiasis is considered a neglected tropical disease (NTD) and mainly affects developing countries where water resources and poor sanitation allow development and infection of snails respectively. It is estimated that around 200 million people are infected worldwide, many of whom live in sub-Saharan Africa. Urinary schistosomiasis is endemic in Mali in general [1]. Both rural and suburban areas are exposed to the parasite through all the country [1,2]. Since 2001, the World Health Assembly endorsed resolution 54.19 which recommends regular de-worming of school-aged children at risk of infection [3,4]. So, control of the infection has gained much international interest when the World Health Organization (WHO) recommended that control of schistosomiasis contributes to the achievement of the Millennium Development Goals (MDGs) [5]. In 2003, more than 75% children and adults living in the eight regions as in the district of Bamako in Mali were treated for schistosomiasis and soil-transmitted helminthiasis through Schistosomiasis Control Initiative (SCI) [4,6]. However, this treatment only targeted school children (6 to 15 years old) and/or adults (over 15 years old) in high-risk occupational groups (e.g. fishermen). Through the changes in population behavior toward water, the epidemiological pattern of schistosomiasis has also changed and many reports are now available on preschool children infection in endemic areas [7-9]. So far, in several control programmes, young children (≤ 6 years old) have been consistently overlooked, in spite of enhanced risk for them to develop morbidity in early childhood.

Thus in order to henceforth justify inclusion of the preschool-aged children in mass treatment programmes, there is the need to obtain more information on the disease. Our study aimed to determine the prevalence and intensity of *Schistosoma haematobium *in the children below 5 years of age in the villages across Diema health district, a rural community with endemic schistosomiasis in north-western of Mali.

## Results

A total of 661 preschool children were screened for this study, but only 338 children submitted urine samples for analysis. Of the sample, 83 were from Fangoune Bamanan, 108 from Dampa and 167 from Debo Kagoro. The age of children ranged from 1-4 years with a mean of 2.90 years. The 2 year-olds included the highest number of preschool children 241 (54.3%). Three hundred and forty-one (53.3%) of them were boys while 276 (44.7%) were girls.

### Prevalence and intensity of *Schistosoma haematobium *infection by sex and age

A total of 173 (51.2%; [95%CI: 45.7-56.2]) children were tested positive for *S. haematobium *infection. Table 1 shows that there was a significant difference in prevalence (p < 0.01) and intensity of infection (p < 0.01) between villages. The overall prevalence of heavy infection was 15.7% while, nearly one-third of the preschool children had light infection (35.5%). Otherwise, there was no significant differences (p = 0.86) in prevalence between the boys and the girls as shown in Table 2. The geometric mean intensity of infection was 18.41 [13.59-24.92] eggs/10 ml of urine. It did not varied significantly between sex (p = 0.91). Preschool children of 4 years old had the highest occurrence in infection with (53.6%) (Table 2). The least occurrence was among those aged 2 years (41.8%). However, there was no significant difference in infection between ages (p = 0.26). Preschool children of 4 years old of age had the highest mean intensity of infection 25.78 [16.55-40.14) eggs/10 ml of urine, while the least 7.11 [3.58-14.12] was in preschool children of 2 years old of age. Thus, the geometric mean egg count increased with age after decreasing in preschool children of 2 years old (p = 0.04) (Table 2).

**Table 1:** Prevalence and intensity of *Schistosoma haematobium *infection of preschool children by village in Diema

			Intensity of infection (% of subjects *¥)*		
Villages	No. Examined	No. infected (%)	Negative (%)	Light (%)	Heavy (%)

Fangoune B	83	70 (84.4)	13 (15.7)	49(40.8)	21 (25.3)

Dampa	108	60 (55.6)	48 (44.4)	39 (36.1)	21 (19.4)

Debo Kagoro	167	44 (26.3)	104 (62.2)	32 (26.7)	11 (6.5)

Total	338	173 (52.1)	165 (48.8)	120 (35.5)	53 (15.7)

P value		0.000			0.000

¥: Negative, light and heavy infections correspond to: 0, 1-49, and ≥ 50 eggs/10 ml urine, respectively.

**Table 2:** Prevalence and geometric mean egg count of *Schistosoma haematobium *infection by sex and age of preschool children in the study villages.

	No. examined	No. infected	Prevalence %	Schistosoma haematobium Geometric mean egg count ¥ (95%CI)
Sex				

Boys	189	98	51.9	17.48 (11.61-26-30)

Girls	149	75	50.3	19.69 (12.42-31.24)

Total	338	173	51.2	18.41 (13.59-24.92)

P value			0.86	0.91

Age				

1 year old	30	14	46.7	15.90 (4.26-59.32)

2 years old	67	28	41.8	7.11 (3.58-14.12)

3 years old	75	42	56.0	17.95 (10.37-30-70)

4 years old	166	89	53.6	25.78 (16.55-40.14)

P value			0.29	0.04

¥: Mann Whitney test for the mean comparison between sex, and Kruskal Wallis test for the mean comparison between age.

### Preschool children water contact exposure

The patterns of water use were similar in all the three study villages. Occupational, bathing, washing, cooking and recreation were the most important activities of water use. FDGs and active observation process of water contact sites revealed that contact of children under three years with the water reservoir was due to their mothers' activities. A mother in Debo Kagoro said: "The lake is the main source of water used by mothers to bathe their children specially those under three years as there is no other source of water". So, older children (4-5 years) admitted that they visit the lake regularly for bathing, washing, swimming and fishing. The FDGs also showed that most of the villagers did not have either a good perception of urinary schistosomiasis as "the presence of blood in urine" or know the precise mode of transmission.

### Treatment and side-effects

During administration of PZQ and ALB in two villages, side-effects directly related to tablet administration (e.g. choking or coughing) were very rare (prevalence < 0.1%) and not life-threatening. Twenty-four hours after treatment in Fangoune Bamanan 3.3% of children (n = 11) reported feeling ill, with abdominal pain (2.1%) and diarrhoea (1.2%) being the commonest symptoms. In the three villages, twenty-hours after treatment, all the side-effects were investigated. Most of side-effects were reported at varying prevalence levels, from 4.6% of children complaining of abdominal pain to 11.7% reporting fatigue. Headache (5.1%), vomiting (9.7%) and diarrhea (10.2%) were found at significant levels (Table 3). By contrast, mothers reported very few children (< 1.0%) with any symptoms present on the day of the 21-days follow-up.

**Table 3:** Percentage (%) of preschool childen (≤ 5 years of age) reported with symptoms 24 hors and 21 days after co-adminsitration of albendazole and praziquantel in the study villages.

Symptoms	Incidence (%)	
	**24 hours later**	**21 days later**

Dizziness	11.3	0.0

Abdominal pain	4.6	1.3

Nausea	8.9	1.0

Diarrhea	10.2	3.5

Vomiting	9.7	2.6

Headache	5.1	3.1

Sleepness	12.8	0.5

Fatigue	7.9	0.6

Lower back pain	0.5	0.0

Urticaria/rash	8.6	7.4

## Discussion

Given our previous observations, we have revealed endemicity of urinary schistosomiasis in populations living closely to lakes across Diema health district [10]. However, several studies on schistosomiasis have tended to focus on school-age and adults, with little or no emphasis on preschool children [11,12]. When preschool children are part of a study, information about them was often subsumed under children aged 6-15 years old and/or adult [13]. The results of this study show a prevalence rate of 51.2% of urinary schistosomiasis among the preschool children in Diema health district. In our study, we observed a high non-compliance rate (51.1%) in urine collection which may be explained by the difficulty of children to give urine between 10.00 AM and 14.00 PM. So, half of the children were not included in the study because of the 10 ml of urine requested, they only have provided less than 5 ml. Another reason that could explain the non-compliance was that 20% of children screened accompanied their mothers out of the village (in fields, lakes, bush etc). The difficulty in obtaining urine samples in this age group led us to request a single sample with known low sensitivity compared to two or more samples which is the best alternatives for accurate diagnosis in this age group. We have preferred a single round of urine sampling to short questionnaires as suggested elsewhere [14] because of the inability of infants to answer correctly questions and the probability that mothers or caregivers could give false answers. So the prevalence we described supports the concept that urinary schistosomiasis was intensively transmitted in Diema health district foci.

In Bandiagara in the north-eastern Mali, the prevalence of *S. haematobium *among preschool children was 5.6% [13]. The prevalence we found was comparable to 52.5% of in a irrigated rice farming community of Kolontomo with single cropping belonging to the vast irrigated rice scheme of Office du Niger. However it was less than 96.2% in settlements of rice farming community of Niono with double cropping in the same perimeter of Office du Niger [15]. The differences in prevalence among these studies could be attributed to types of water bodies and water contact practices. All the ages studied were infected, meaning that infection with urinary schistosomiasis occurs very early in life through exposure to contaminated water bodies either by guardians and caregivers or the preschool children themselves. As the lakes were the only source of water supply for people and animals in the three study communities, it is difficult if not impossible to prevent communities from visiting these water bodies everyday for various needs.

Nevertheless, there was no significant difference in the prevalence between boys and girls (p = 0.86) as well as age (p = 0.29). This may be an indication that both gender and age are equally exposed to infection through water contacts. Contacts with the ponds by preschool children remain abated throughout their preschool age. The mean intensity of infection reported here is higher than those reported elsewhere [16,17]. The reason for this difference could be due to several factors such as frequency of water contacts, and density of infected snail in the water bodies, especially during the hot hours of the day. Even if no difference in the prevalence of infection between age, the intensity of infection significantly increased with age (p = 0.04). This means that preschool children of 4 years old of age were intensively and permanently exposed to cercariae in the water bodies than those of 1 year or 2 years old of age. To support this observation, preschool children of 3-4 years old admitted they visit lakes every day for washing, bathing and swimming with any assistance of their parents. The FDGs revealed that most of villagers were aware of the health risks of lakes particularly at the end of dry season (April and June), yet they lack the capacity to change their behavior, as water was cloudy and stagnant at this period.

For preschool aged children, early exposition to infection could lead to early complications compared to late infection, if left treatment. Involving communities in repairing their safe water source could help reduce the contact with water bodies and transmission of the disease. However, the FGDs showed that a safe water supply may not reduce contact with the lakes because most water contacts are either recreational or occupational, and a new water supply might at best be used for domestic needs. The mothers or guardians and caregivers should also be enlightened about the avoidance of the lakes with their children by heating the water before using it to bathe and not taking the children along with them to the water bodies [18]. So, any control measure must involve the community [19], such as developing participatory health education programmes with community members to effect behavioral change by mothers and by caregivers who expose their young children to schistosome infection and to understand disease transmission. High temperature and sun exposure promote cercariae production into water by snails between 11:00 AM to 15:00 PM, which increase significantly infection rate within population. Several authors call for inclusion of infant and preschool children for treatment in schistosomiasis control programmes in endemic areas [18,20,21]. Although, we treated all preschool children tested positive for schistosomiasis with the WHO standard recommended dose 40 mg/kg of praziquantel. There was no significant difference of the side effects reported here and those observed classically [16]. So, administration of PZQ in the young child resulted in some putative side-effects, although many had resolved by 24 hours after treatment and even fewer were present 21 days after treatment. However, the issue of the safety of praziquantel in infants less than 5 years old needs to be addressed as seen in a recent study where side effects were reported [22]. As reported by these authors in Ugandan and Zanzibari children, further studies are needed in other endemic settings on the safety of PZQ treatment in infants and its extended dose pole to this age group. This challenge could be solved using new PZQ syrup formulation at low cost, delicious and easy to be administered in infants than tablets.

## Conclusion

The emerging fact from the study shows that preschool children also harbor schistosomiasis infection and are therefore also source of transmission in endemic communities. Excluded in the past from any control programme, provision of their inclusion in treatment programmes is imperative due to the risk to develop early severe forms of the disease and the possibility to transmit parasites.

## Materials and methods

### Study areas and population

**Figure 1:**
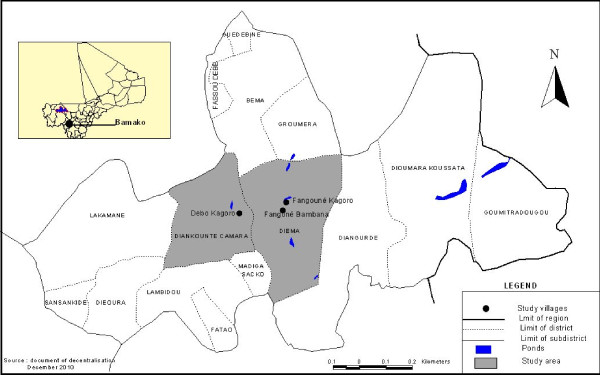
**Localization of study villages (black circle) in the district of Diema**.

Diema is a district (latitude 009°12.834N and longitude 14°36.594E) and approximately 216 kilometers from Kayes, a region capital of Mali. The study took place in three villages (Fangoune Bamanan, Dampa and Debo Kagoro) from 7 to 10 kilometers around Diema (Figure 1). Members of these communities are farmers and traders of Soninke and Bambara ethnic groups. In Diema health district, the main sources of water used by the population are large lakes. These lakes are used not only for domestic activities (bathing, drinking, washing, recreation and cooking) but also as a drinking water for animals. None of water supplies installed either by the populations or by nongovernmental organization (NGO) was working. So, even the sources for water supply are functioning well, the population did not use them unless the lake dried. With help from teenagers, we were able to identify all sites of water contact. These sites are used for bathe as well as areas of distraction for children. Besides the lakes, there are also, some large diameter wells and water pump systems. There is no health center in the villages. All the children below 5 years of age were registered in the three communities after a house-to-house census. An epidemiological form was used to register children which documented their age, sex, and the name of the household head. Due to the small number of preschool children in the community, sampling and sample size calculation were not necessary. All the children who provided urine samples for examination were enrolled in the study.

### Urine collection

As the study was focused on young children, each child's mother or guardian was requested for assistance in provision of urine samples from her child. Sterile plastic containers (labeled) were given to the parents/guardians of the children to collect urine samples. The urine collected was then immediately taken to the laboratory for analysis. A total of 661 children were screened, but only 338 children provided urine samples.

### Examination of *Schistosoma haematobium *ova

For parasitological diagnosis of *S. haematobium*, a single urine sample was collected between 10.00 am and 14.00 pm hours from each child; the number of eggs was counted and expressed as egg/10 ml urine. The urine was strained through filters (Nucleopore, Action, MA) with Whatman (Brentford, United Kingdom) filter paper (22 μm). Filter papers were stained with 5% ninhydrin and examined for presence of *S. haematobium *eggs when dry. The intensity of infection was expressed as negative (0 egg/10 ml urine), light (1-49 eggs/10 ml urine) and heavy (≥ 50 eggs/10 ml urine). Chemotherapy campaigns administering praziquantel (PZQ) for schistosomiasis and albendazole (ALB) for soil-transmitted helminthiasis (STHs) are now the front-line intervention to prevent severe and permanent disabilities in endemic areas.

Based on this control strategy, each child, regardless of presence of *S. haematobium *egg was treated with praziquantel tablets (40 mg/kg) as recommended by WHO (1985) [23]. For younger children (under 2 years old), the PZQ was crushed and mixed with water before administration (spoon-feeding), with the help of the mother, with a half-tablet of ALB. To describe side effects associated to PZQ administration, a follow-up of all the 338 preschool children was performed 24 hours and 21 days after treatment, where mothers were asked to report any of the following symptoms since treatment: dizziness, headache, sleepiness, fatigue, vertigo, abdominal pain, cramps, nausea, vomiting, diarrhea, bloody stools, lower back pain and urticaria/rash. Mothers also were invited to respond on behalf of their children to whether they experienced any of the stated symptoms prior to treatment, at 24 hours and 21 days after-treatment.

### Water contact practices

At first, we held focus group discussions (FGDs) and in-depth interviews with the community members (mostly the mothers and/or guardians of children) concerning water contact practices in each village. The sessions with preschool children were limited to children between 4 and 5 years of age. We got information about children under 4 years of age by conducting two others sessions with adult males and adult females (mostly nursing mothers). Then, with the assistance of teenagers, we visited during seven consecutive days some water contact sites to observe actively how preschool children were exposed to cercariae.

### Statistical analysis

Data were double entered using Epi-Info version 6.04 (CDC, Atlanta, GA, USA) and analyzed using STATA 9.1. Differences in proportions were tested using chi-square, either for trend or for independence, as appropriate. For egg mean counts, the geometric means of Williams, GMw, was chosen as the measure of central tendency due to the typical over-dispersion present in this type of data (Mann Whitney test for the mean comparison between sex, and Kruskal Wallis test for the mean comparison between age), and CI_95 _values for GMw were estimated according to Kirkwood and stern.

### Ethical approval

Before the study began, the village heads together with guardians and caregivers were fully briefed on the objective of the study. Thereafter, the guardians and caregivers were given an informed consent form to sign for their children, after its content was translated to them in local language. Only preschool children whose guardian and caregivers signed (or fingerprinted) the consent sheet participated in the study. The study objective was also explained to older preschool children for their understanding and cooperation. The protocol study was reviewed and approved by the ethical review board of the Faculty of Medicine and Dentistry of Bamako (Mali).

## Competing interests

The authors declare that they have no competing interests.

## Authors' contributions

AD has participated in the conception and design of the study, data analysis and interpretation. He also contributed in the writing of the manuscript and assured the coordination of the trial. He has reviewed the final version. BB participated in design of the study and in on-site execution by collecting and analyzing data. He also had all the clinical responsibility and supervised drug distribution coordinated the fields' activities. HMB participated in on-site execution, assessment of side effects, data analysis and interpretation. He also contributed in drug distribution. OKD participated in the conception and design of the study. He contributed in the writing of manuscript, data analyzing and participated in drafting the paper and reviewed the final version. All the authors read and approved the final manuscript.
